# Efficacy of Onabotulinum Toxin A (Botox) versus Abobotulinum Toxin A (Dysport) Using a Conversion Factor (1 : 2.5) in Treatment of Primary Palmar Hyperhidrosis

**DOI:** 10.1155/2013/686329

**Published:** 2013-10-22

**Authors:** Hanan Mohamed El Kahky, Heba Mahmoud Diab, Dalia Gamal Aly, Nehal Magdi Farag

**Affiliations:** ^1^Department of Dermatology and Venereology, Ain Shams University, Abasseya Square, Cairo, Egypt; ^2^National Research Center, El-Tahrir Street, Dokki, Giza 12622, Egypt

## Abstract

*Background*. Two preparations of botulinum A toxin (BTX-A) are commercially available for the treatment of palmar hyperhidrosis (PPH): Botox (Allergan; 100 U/vial) and Dysport (Ipsen Limited; 500 U/vial), which are not bioequivalent. Results regarding an appropriate conversion factor between them are controversial. *Objectives*. This paper aims to compare the efficacy of Botox and Dysport in PPH using a conversion factor of 1 : 2.5. *Methods*. Eight patients with severe PPH received intradermal injections of Botox in one palm and Dysport in the other in the same session. Clinical assessment was performed at baseline and posttreatment for 8 months using Minor's iodine starch test, Hyperhidrosis Disease Severity Scale (HDSS), and Dermatology Life Quality Index (DLQI) test. *Results*. At 3 weeks, a significant decrease in sweating for both preparations was noted which was more pronounced with Dysport compared with Botox. At 8 weeks, this difference turned insignificant. Continued evaluation showed similar improvement in both palms with a nonsignificant difference. Patients with longer disease duration were more liable to relapse. *Conclusion*. The efficacy and safety of Botox and Dysport injections were similar using a conversion factor of 1 : 2.5. There was a trend towards a more rapid action after Dysport treatment but without significant importance.

## 1. Introduction

Palmar hyperhidrosis (PHH) is a condition characterized by excessive sweating of the palms. The condition causes significant disruption in both social and professional life, leading to a marked impact on the patient's quality of life (QOL) [1].

Botulinum toxin is a potent neurotoxin which specifically inhibits the release of acetylcholine from nerve terminals by acting mainly on the cholinergic synapses [2]. It has been well studied in neurology as well as other disciplines, as focal hyperhidrosis, axillary, and PHH and is considered the treatment of choice in many of its indications [3].

Although the human nervous system is affected by all seven types (A–F), if applied parentally, type A preparations are most commonly used in clinical practice for various reasons such as their prior availability, immunologic aspects, safety, and efficacy [4]. Among the type A preparations, are Onabotulinum toxin A or Botox (Allergan, Inc., Irvine, CA) and Abobotulinum toxin A or Dysport (Ipsen Limited, Slough, Berkshire, UK) which are not bioequivalent [5]. Botox is purified by repeated precipitation and redissolution and is formulated with 500 mg human serum albumin with 0.9 mg sodium chloride. It contains less than 5 ng of Botulinum toxin A (BTX-A) in a 900 kDa complex [6]. Whereas Dysport is produced by purification using column separation method yielding a mixture of complex-sized toxin proteins and contains 125 mg human serum albumin with 2.5 mg lactose [6, 7]. Moreover, it contains 12.5 ng of BTX-A in a multiprotein preparation ranging from 500 to 900 kDa [8]. 

The variation between both Botox and Dysport led to a controversy about their respective potencies and their diffusion characteristics [4].

Different studies have been done to find an appropriate conversion factor, but the results are conflicting. There is still no official consensus regarding the ideal dosage conversion factor between the two agents [9–11]. However, taken together, the findings retrieved from the literature research suggest that a conversion ratio of 1 : 3 (Botox : Dysport)—or even lower—can be considered appropriate. Higher conversion ratios may lead to an overdosing of Dysport, with a potential increased incidence of adverse events [4, 9]. Therefore, the aim of this study was to investigate the use of a conversion factor between Botox and Dysport of 1 : 2.5 in PHH and to compare the effect of this dilution on the efficacy and tolerability of each preparation.

## 2. Patients and Methods

### 2.1. Patients

This study included eight patients (five men and three women) with severe PHH since childhood. All patients experienced impairment in the QOL and had not responded to any conventional topical and physical treatments (e.g., local antiperspirants containing aluminium salts, application of acids or aldehydes, and iontophoresis). Patients were excluded if they were prepubertal, pregnant, or breast feeding women, with any neuromuscular diseases or systemic diseases that could cause secondary hyperhidrosis, and those using systemic medications that could interfere with neuromuscular activity (e.g., aminoglycosides). 

All patients were recruited from the Dermatology out-patient clinics of the National Research Centre and Ain Shams university hospitals. All of them gave an informed consent to participate in this work. The study was approved by the research ethics committee of the National Research Center, Giza, Egypt. 

### 2.2. Methods

The study followed a single-blind, right-left comparative design. Each patient received a total dose of 50 U of Botox in the right palm and 125 U of Dysport in the left palm, in the same session. All patients were subjected to detailed history taking and clinical assessment of the hyperhidrotic area at baseline and posttreatment at 3, 8, and 13 weeks, as well as 6 and 8 months. Photographic documentation (Panasonic LZ8 camera, 8.1 megapixel, China) for each patient before and after the course of treatment was done to compare and evaluate the clinical response and side effects of the treatment. Classification of disease severity, identification of hyperhidrotic area extension, and evaluation of patients' QOL improvement were done.

## 3. Measurement of Disease Severity

The Hyperhidrosis Disease Severity Scale (HDSS) was chosen to measure the disease severity [12]. A score of 3 or 4 indicated severe hyperhidrosis.

## 4. Evaluation of Palm Sweating Area (PSA) 

Visualization of the extension of hyperhidrotic area was obtained by the Minor-iodine starch test. Iodine solution was spread on the skin. Starch powder was then applied to achieve good colorimetric response. The sweat production then caused the mixture to turn into dark blue, making it easy to identify the extent of sweating as follows: Score (0) (no involvement, yellow without dark blue); Score (1) sweating area <25% of target zone; score (2) sweating area between 25% and 50% of the target zone; score (3) sweating area between 50% and 75% of the target zone; score (4) sweating area >75% of target zone [13]. A digital photograph of both hands was taken 5 minutes after the application of the starch powder.

## 5. Evaluation of Patients' Quality of Life

A subjective evaluation of the disability caused by the symptoms of hyperhidrosis was obtained using the Dermatology Life Quality Index (DLQI) [14] which is a validated tool that demonstrates changes after skin disease treatments. It consists of 10 questions regarding work, leisure, daily activities, personal relationships, and treatments. Each question has five alternative answers with corresponding scores. The higher the score, the greater was the impairment of QOL.

## 6. Intradermal Injection of Botulinum A Toxin

Topical anesthesia was applied before treatment on both palms, under occlusion, for 60 minutes to minimize the pain of the injections. Both the Botox vial (100 MU/vial) and the Dysport vial (500 MU/vial) were reconstituted with 5 mL of 0.9% sterile saline to obtain a concentration of 2 MU/0.1 ml of Botox and 10 MU of Dysport/0.1 ml, respectively. By this reconstitution, each 2 MU of Botox was equivalent to 5 MU of Dysport using a conversion factor of 1 : 2.5. Botulinum A toxin was intradermally injected in each palm in 25 different sites (i.e., 2 MU of Botox/site in one palm and 5 MU of Dysport/site in the other palm) using a 27-gauge needle.

## 7. Subjective Evaluation of Side Effects

Patients were instructed to report any side effects as regards type, duration, and severity after the injection session and at every follow-up visit. Patients were also questioned regarding the presence of pain in the area of the injections, bruising, and reduction in muscular force in the hand or thumb grip and/or compensatory sweating in other sites. 

## 8. Statistical Analysis

Data were analyzed using Statistical Package for Social Science (SPSS) version19.0 for windows; IBM Corporation, New York, August 2010. Mean and standard deviation were used to describe continuous data, number, and percentage for categorical data. Comparison between two means was done using an independent sample Student's *t*-test. Paired *t* test was used to assess the statistical significance of the difference between two means measured twice for the same study group. Chi-Square test was used to compare between two qualitative variables while Correlation Analysis (using Pearson's method) was used to assess the strength of association between two quantitative variables. The correlation coefficient denoted symbolically as “*r*” defines the strength and direction of the linear relationship between two variables. The McNemar test was used to assess the statistical significance of the difference between a qualitative variable measured twice for the same study group. *P* values < 0.05 were considered statistically significant.

## 9. Results

Of the 8 patients included in the study, 5 were males (62.5%) and 3 were females (37.5%). Their ages ranged from 16 to 31 years with a mean ± SD of 23.1 ± 5.7 years. All patients had severe hyperhidrosis. Both palms were equally affected in 6 patients (75%), whereas 2 patients (25%) had their left palm slightly more affected without any significance between both (*P* value >0.05). All patients had hyperhidrosis since childhood, with an age of onset between 6 to 10 years with a mean ± SD of 7.8 ± 1.3 years. Disease duration ranged from 9 to 22 years with a mean ± SD of 15.4 ± 5years. Family history was positive in 2 patients (25%) and was negative in 6 patients (75%). Stress and physical or psychological impairment were the most significant aggravating factors reported by all patients. Secondary changes were associated with the disease in all patients where 5 patients (62.5%) had fissuring, 2 (25%) had warts and burning sensation, and 1 (12.5%) had paronychia.

## 10. Comparison between Baseline Values

There were no substantial differences found at baseline between the Botox- and Dysport-treated palms, as regards the extent of sweating. The mean ± SD PSA in the Botox versus Dysport treated-palms was (3.25 ± 0.46 versus 3.5 ± 0.53 resp., *P*-value >0.05). Both palms had a similar number of injection sites (25 injection site). The mean ± SD dose injected per palm was 50 ± 6 U Botox, and 125 ± 15 U Dysport, respecting the 1 : 2.5 conversion ratio.

Additionally, no correlation was found at baseline between the severity of the disease in the Botox versus the Dysport-treated palms with the age of patients (*r* = −0.17 versus −0.12, resp., *P* value >0.05), age of onset of the disease (*r* = 0.12 versus 0, resp., *P* value >0.05), disease duration (*r* = −0.23 versus −0.13, resp., *P* value >0.05), or family history of patients being positive or negative (*r* = 0.63 versus 0, resp., *P* value >0.05).

## 11. Comparison between Palm Sweating Area Score in the Botox- and Dysport-Treated Palms at Different Follow-Up Visits

Minor-iodine starch test revealed a highly significant reduction in the PSA score in all patients, after injection of both preparations during the follow-up visits (at 3 weeks, 8 weeks, 13 weeks, 6 and 8 months), compared to PSA 0 score at baseline, (*P* value = 0.0001) (Tables 1 and 2). However, on comparing both palms together a nonsignificant difference was noted (*P* value >0.05).

**Figure 1:**
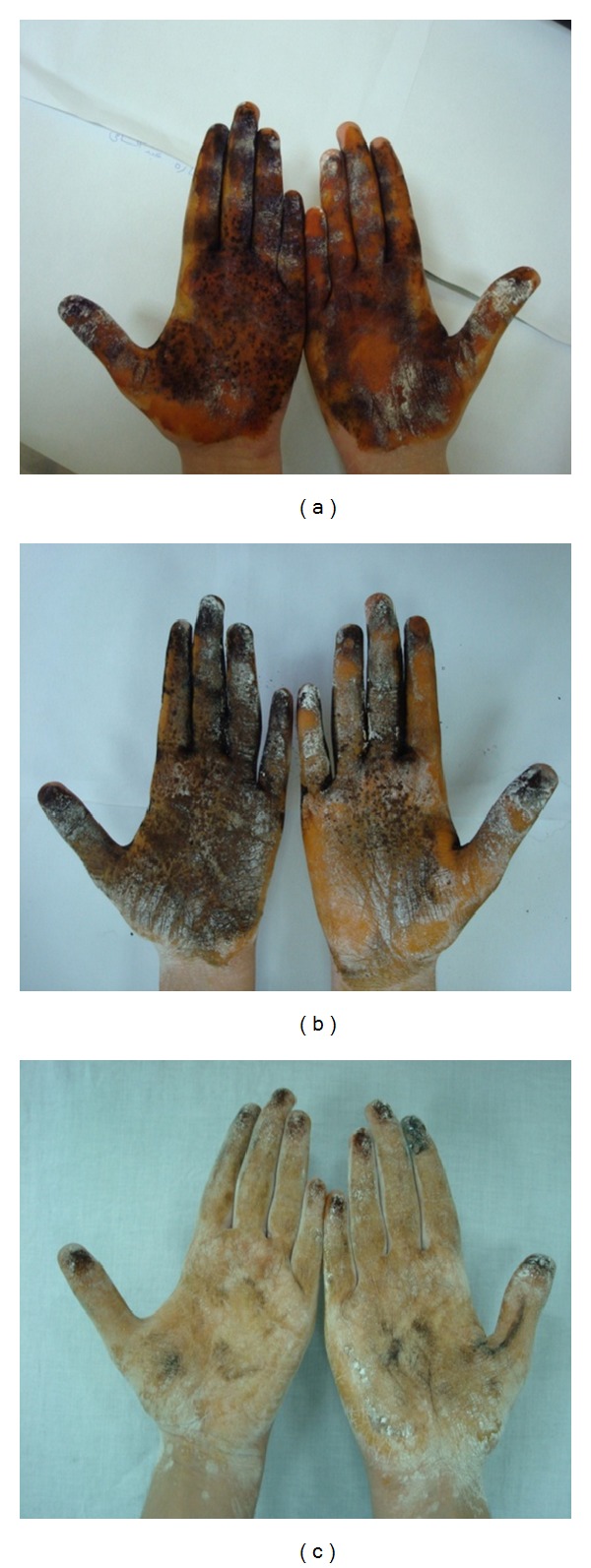
Right hand treated with Botox, left hand with Dysport showing Minor's iodine starch test (a) before treatment, (b) 3 weeks posttreatment, and (c) 8 weeks posttreatment.

Of special note, at 3 weeks posttreatment, there was a significant decrease in the mean PSA of baseline sweating area being more in all the Dysport-treated palms compared to all the Botox-treated palms (Mean ± SD of PSA score was 1.38 ± 0.52 versus 2 ± 0, resp., *P* value <0.05). Meanwhile, at 8 weeks posttreatment, the PSA score was equal in both palms with a mean ± SD of 1 ± 0 (Figure 1). Minor differences in the PSA score between both palms were detected at 13 weeks, 6 and 8 months posttreatment, that did not reach any statistically significant values (*P* value >0.05).

Patients were followed up for 8 months after the injection of BTX-A for recurrence using the PSA score. Each change in the PSA score was considered 25% recurrence. Three patients (37.5%) after 8 months complained of 25% relapse in their right palm (injected with Botox), while 4 patients (50%) after 8 months complained of 25% relapse in their left palm (injected with Dysport), but there was a non significant difference between the 2 palms (*P* value >0.05). Relapse was directly related to disease duration, as patients with longer disease duration were more liable to relapse (*P* value <0.05). However, it was not influenced by the age or sex of the patients. There was also no statistically significant relation between it and the age of onset of the disease or the family history of the disease (*P* value >0.05).

## 12. Assessment of Quality of Life Improvement after Treatment

All patients showed an increase in their QOL after treatment with either Botox or Dysport with a nonsignificant difference between them. The lower the DLQI, the better was the QOL. The mean ± SD of DLQI before treatment was 81.8 ± 13.8 decreasing to 18.1 ± 7.9 after treatment, indicating a highly significant difference after treatment (*t* = 9.87, *P* value <0.05). 

## 13. Side Effects

Five patients (62.5%) reported weakness in the form of mild decrease in the hand grip, in both palms which lasted for 3 weeks and resolved without any residual effect, while 2 patients (25%) had tingling and numbness that lasted for about 3 weeks and resolved spontaneously. 

## 14. Discussion

A strong controversy still remains about the conversion ratios between Botox and Dysport. Data in the literature ranged between 1 : 2.2 and 1 : 11 [4]. Initial conversion recommendations, dating back about 15 years to Dysport's European approval, suggested a Botox : Dysport ratio of 1 : 5 or 1 : 4 [15]. Today, many clinicians use a 1 : 3 conversion factor [4, 16]. However, several authors suggested that such ratios may be high and there was a consistent trend in most of those studies for Dysport treatment to have more adverse events and longer lasting effects [17]. One publication placed the conversion ratio at 1 : 1.3–1.6 and reported that both preparations were similar in the efficacy and in the diffusion characteristics [4]. Others suggested that ratios as 1 : 2.5 may be more accurate [18]. Additionally, in Germany, the manufacturers recommend the Botox: Dysport conversion ratio to be 1 : 2.5 and in Austria, both products are registered for the treatment of axillary hyperhidrosis, with an initial dose of 50 U Botox or 100 U Dysport (i.e., a ratio of 1 : 2) [17]. Accordingly, in the current work, we decided to use a conversion factor between Botox and Dysport of 1 : 2.5 U in treating PPH and to study the efficacy of this ratio in bringing out the desirable effect with a less potential than doses with ratios 1 : 4 and 1 : 3. 

Palmar hyperhidrosis represents a good model to perform such Botox/Dysport comparative studies in which each patient acts as his/her own control. The application of two preparations of toxin in the same patient, one in each palm, was considered appropriate because it eliminated several important factors, such as the difference in the quantity of sweat produced by one individual in comparison to another, possible variations in temperature, and physical and psychological alterations that may lead to variation in the sweat production [19].

Our findings of similar levels of efficacy and safety for both Botox and Dysport with a conversion ratio of 1 : 2.5 U is in line with those of Hexsel et al. (2008) [20] who reported that an equivalence ratio of 1 : 2 or 1 : 2.5 U resulted in similar results, in terms of safety and efficacy, whereas higher equivalences increase the risks of side effects related to the potency. Our data as well as those of Hexel and colleagues suggest that clinically the preferred conversion ratio is less than 1 : 3. In contrast to our findings, several commercially sponsored studies claimed considerably higher conversion ratios [11, 21].

Beyond the controversy about the respective conversion ratios, there is also considerable debate on the diffusion characteristics of the two products [22, 23]. Kranz et al. (2009) [4] reported similar diffusion characteristics for both agents in their human skin model test of Botox and Dysport. Some data and reports of clinical experience suggest that Dysport may diffuse more readily than Botox does and that it has more side effects than Botox due to a higher diffusion rate [24]. Different pharmaceutical properties of the two products as complexing proteins or excipients have been suggested to cause these diffusion differences [23].

Our findings of a highly significant decrease in the PSA score after treatment in both palms of all patients being more with Dysport as compared to Botox in the 1st follow-up visit (3 weeks) and then the similarity of the PSA score in both palms in the rest of all follow-up visits could be explained by the fact that Dysport diffuses more quickly than Botox, as it has a lighter molecular weight than Botox (Dysport 400 kDa versus 900 kDa in Botox), resulting in an earlier effect as early as three days after injection [5]. Furthermore, we speculated that when Botox reached its full therapeutic effect in the 2nd follow-up visit (8 weeks after injection), it gave the same therapeutic effect as Dysport in the other follow-up visits. Pickett (2009) [25], however, disagrees stating, “Diffusion of botulinum toxin products is not related to the size of the toxin complex in the product since the complex dissociates under physiological conditions, releasing the naked neurotoxin to act.” This in our opinion may become an issue settled through cumulative clinical experience rather than pure science.

The side effects associated with BTX-A injection include, for example, injection site reactions and muscle weakness which tend to be transient and are not specific to any formulation [26]. In the present study, 50 U Botox and 125 U Dysport had very mild and tolerable effect on the hand grip weakness in 37.5% that resolved within 3 weeks spontaneously. Others experienced some tingling and heaviness that lasted for few days and resolved without any medical intervention on both hands. These complications being transient could be mainly due to the pain the patients suffer during the multiple site injection procedure. Higher rates of temporary weakness have been reported when Botox or Dysport is applied subcutaneously; thus, the intradermal injection technique, as used in this study, is recommended [3].

We want to emphasize the statements that Dysport tends to have higher efficacy, longer duration, and higher frequency of adverse events than Botox as published by Simonetta Moreau et al. (2003) [15] and Kranz et al. (2009) [4] which indicate that most head-to-head trials comparing the two products used too high conversion ratios compared to our study where a conversion ratio of 1 U Botox: 2.5 U Dysport was used. 

In this study, we also analyzed the role that some clinical factors could play in predicting the duration of therapeutic response as they may have an important prognostic value. Sex, age of patients, age of onset of the disease, and family history did not influence the relapse-free period indicating that these variables could not be considered as predicting factors of response to BTX-A therapy. On the other hand, the relapse-free period was affected significantly by the disease duration. Relapse occurred earlier in patients with longer disease duration, which is in accordance with a recent study that stated the same fact [13]. Disease duration seems to be a clinical factor predictive of response to therapy with BTX-A. We therefore believe that it is better to advice patients to start treatment early for longer relapse-free period.

The finding that relapse started to appear in 4 patients in both hands after 8 months of treatment but did not reach any significant importance indicates that the effect of BTX-A in PPH may last for more than 8 months. This duration is in accordance with other studies in the literature [2, 3]. However, it was longer than other studies who stated that the effect of BTX-A injection lasted for only 2 months [15]. Nevertheless, we believe that longer follow-up periods are required in future studies to have more precise and demonstrative findings.

Of special note, the severity of the disease in this work was not related to patients' demographic data as age, sex, age of onset of the disease, or family history being positive or negative. Patients reported stress to be the most common aggravating factor for their PPH. Stress is known to increase the levels of ACh and hence increases stimuli of the sweat glands causing more sweat production [27, 28]. Secondary skin changes in the form of fissuring, warts, burning sensation, and paronychia were reported by all patients to be associated with their PPH. Wet hands cause fissuring, thus decreasing the natural skin barrier, making the patient more prone to external bacterial and viral infection [6].

To sum up, on the basis of this work, we suggest that lower conversion ratios are to be used when treating PHH as Botox and Dysport presented similar levels of efficacy and safety during the 8-month follow-up when a conversion factor of 1 : 2.5 was used. There was a trend towards a greater improvement after Dysport treatment initially but it did not reach statistical significance. Disease duration was found directly related to the occurrence of relapse; thus treatment should be started early to get better outcomes with longer periods of cure. Longer follow-up period is however recommended to confirm or refute our findings.

## Figures and Tables

**Table 1:** Comparison between palm sweating area (PSA) score before treatment and at follow-up visits in the right palm (Botox).

	Mean ± standard deviation	*t* test	*P* value	Significance
Right PSA 0	3.2 ± 0.4	—	—	—
Right PSA 3 weeks	2 ± 0	7.6	0.0001	HS
Right PSA 8 weeks	1 ± 0	13.7	0.0001	HS
Right PSA 13 weeks	0.5 ± 0	14.7	0.0001	HS
Right PSA 6 months	0.8 ± 0.5	12.9	0.0001	HS
Right PSA 8 months	1 ± 0.6	10.9	0.0001	HS

HS: highly significant.

**Table 2:** Comparison between palm sweating area (PSA) score before treatment and at follow-up visits in the left palm (Dysport).

	Mean ± standard deviation	*t* test	*P* value	Significance
Left PSA 0	3.5 ± 0.5	—	—	—
Left PSA 3 weeks	1.3 ± 0.5	17	0.0001	HS
Left PSA 8 weeks	1 ± 0	13.2	0.0001	HS
Left PSA 13 weeks	0.2 ± 0.5	17.9	0.0001	HS
Left PSA 6 months	0.3 ± 0.4	15.9	0.0001	HS
Left PSA 8 months	0.7 ± 0.7	8.7	0.0001	HS

HS: highly significant.
